# Novel Antimicrobials for the Treatment of *Clostridium difficile* Infection

**DOI:** 10.3389/fmed.2018.00096

**Published:** 2018-04-16

**Authors:** Nicola Petrosillo, Guido Granata, Maria Adriana Cataldo

**Affiliations:** Clinical and Research Department for Infectious Diseases, Unit Systemic and Immunedepression-Associated Infections, National Institute for Infectious Diseases L. Spallanzani, Rome, Italy

**Keywords:** *Clostridium difficile* infection, novel antimicrobials, *Clostridium difficile* recurrence, prevention, management

## Abstract

The current picture of *Clostridium difficile* infection (CDI) is alarming with a mortality rate ranging between 3% and 15% and a CDI recurrence rate ranging from 12% to 40%. Despite the great efforts made over the past 10 years to face the CDI burden, there are still gray areas in our knowledge on CDI management. The traditional anti-CDI antimicrobials are not always adequate in addressing the current needs in CDI management. The aim of our review is to give an update on novel antimicrobials for the treatment of CDI, considering the currently available evidences on their efficacy, safety, molecular mechanism of action, and their probability to be successfully introduced into the clinical practice in the near future. We identified, through a PubMed search, 16 novel antimicrobial molecules under study for CDI treatment: cadazolid, surotomycin, ridinilazole, LFF571, ramoplanin, CRS3123, fusidic acid, nitazoxanide, rifampin, rifaximin, tigecycline, auranofin, NVB302, thuricin CD, lacticin 3147, and acyldepsipeptide antimicrobials. In comparison with the traditional anti-CDI antimicrobial treatment, some of the novel antimicrobials reviewed in this study offer several advantages, i.e., the favorable pharmacokinetic and pharmacodynamic profile, the narrow-spectrum activity against CD that implicates a low impact on the gut microbiota composition, the inhibitory activity on CD sporulation and toxins production. Among these novel antimicrobials, the most active compounds in reducing spore production are cadazolid, ridinilazole, CRS3123, ramoplanin and, potentially, the acyldepsipeptide antimicrobials. These antimicrobials may potentially reduce CD environment spread and persistence, thus reducing CDI healthcare-associated acquisition. However, some of them, i.e., surotomycin, fusidic acid, etc., will not be available due to lack of superiority versus standard of treatment. The most CD narrow-spectrum novel antimicrobials that allow to preserve microbiota integrity are cadazolid, ridinilazole, auranofin, and thuricin CD. In conclusion, the novel antimicrobial molecules under development for CDI have promising key features and advancements in comparison to the traditional anti-CDI antimicrobials. In the near future, some of these new molecules might be effective alternatives to fight CDI.

## Introduction

The Gram-positive, anaerobic, spore-forming bacillus *Clostridium difficile* (CD) represents the most common cause of nosocomial diarrhea worldwide (1–5). According to literature data, a total of 15%–25% of all cases of antibiotic-associated diarrhea result from *C*. *difficile* infection (CDI) (1–5).

The current picture is alarming with a CDI mortality rate ranging between 3 and 15% and a CDI recurrence rate ranging from 12 to 40% (6–8). Importantly, after the first recurrence, a risk up to 64% of subsequent recurrences has been reported (6–10).

Despite the great efforts made over the past 10 years to face the CDI burden, there are still gray areas in our knowledge on CDI management. Major issues affecting the management of CDI include the high rate of CDI underdiagnosis and the delay in diagnosing it, the unacceptably high rate of CDI recurrence, and the difficulties faced in reducing the spread of CD among hospitalized patients (11–15).

Recurrences currently represent one of the major challenges in the management of CDI, resulting in higher hospitalization costs and in increased morbidity and mortality rate (16). Of relevance, semi-structured interviews with patients who had experienced CDI showed that this disease affects numerous aspects of patients’ lives and causes patients’ emotional distress (17).

The therapeutical management of CDI is mainly based on discontinuation of unnecessary antibiotics and administration of anti-CD antimicrobials (18).

The currently recommended first-line antimicrobial therapy is represented by oral metronidazole or oral vancomycin for the first episode of mild CDI, and oral vancomycin for severe CDI or subsequent CDI recurrences (13, 18, 19). Oral fidaxomicin is also a treatment of choice for recurrent CDI, especially in those with a high risk of relapse (13, 18, 19).

Metronidazole and vancomycin achieve an end-of-treatment cure rate of approximately 86–95% (19, 20); however, they are not as much effective in assuring sustained and bacteriological cure, defined as the prevention of recurrent CDI and the prevention of CD spread, respectively (20–22).

Recently new innovative approaches, based on non-antimicrobial compounds, i.e., monoclonal anti-toxin antibodies, fecal microbiota transplantation, live bacterial vaccines and CD vaccines, have been developed or are under development. However, further studies are needed to confirm the efficacy and safety of these approaches.

Moreover a number of new agents active against CD have been developed and their use for CDI treatment is under study, and hopefully in the near future, these new antimicrobials will represent effective options to fight CDI.

Finally, old antimicrobial agents approved for the treatment of other infection showed activity against CD, and their utility for CDI is now being studied.

The aim of our review is to give an update on the novel antimicrobials for the treatment of CDI, considering the currently available evidences on their efficacy, safety, molecular mechanism of action, and their probability to be successfully introduced into the clinical practice in the near future.

## Materials and Methods

### Search Strategy and Selection Criteria

Through a PubMed search with the search terms “novel antimicrobials AND clostridium difficile” and “antimicrobial treatment AND clostridium difficile,” we identified 16 novel antimicrobials for the treatment of CDI: cadazolid, surotomycin, ridinilazole, LFF571, ramoplanin, CRS3123, fusidic acid, nitazoxanide, rifampin, rifaximin, tigecycline, auranofin; NVB302, thuricin CD, lacticin 3147, and acyldepsipeptide antimicrobials.

Published articles from January 2000 to November 2017 reporting the use of these 16 antimicrobials for the treatment of CDI in human patients were identified through computerized literature searches using MEDLINE (National Library of Medicine Bethesda MD) and by reviewing the references of retrieved articles.

Indexed search terms included: “cadazolid AND clostridium difficile” OR “ACT-179811 AND clostridium difficile” OR “Surotomycin AND clostridium difficile” OR “CB-183315 AND clostridium difficile”; “Ridinilazole AND clostridium difficile” OR “SMT19969 AND clostridium difficile” OR “LFF571 AND clostridium difficile” OR “Ramoplanin AND clostridium difficile” OR “CRS3123 AND clostridium difficile” OR “REP3123 AND clostridium difficile” OR “fusidic acid AND clostridium difficile” OR “nitazoxanide AND clostridium difficile” OR “rifampin AND clostridium difficile” OR “NVB302 AND clostridium difficile” OR “thuricin CD AND clostridium difficile” OR “lacticin 3147 AND clostridium difficile” OR “auranofin AND clostridium difficile” OR “acyldepsipeptide AND clostridium difficile” OR “tigecycline AND clostridium difficile” OR “rifaximin AND clostridium difficile”.

No attempt was made to obtain information about unpublished studies. English language restriction was applied.

## Results

Our literature search identified 453 studies, an additional 4 articles were identified by reviewing the references of retrieved articles.

Regarding cadazolid, we identified 20 studies, 3 of them were clinical trials: a phase II clinical trial with the main objective to evaluate the susceptibilities of CD isolates to cadazolid and vancomycin (23); a multicenter, randomized, double-blind, phase II trial comparing the clinical cure rate of cadazolid versus vancomycin at 48–24 h after the end of treatment (24); and a single-center, open-label, single oral dose phase I study to investigate systemic cadazolid exposure (25).

Regarding surotomycin, our literature search identified 30 studies, 6 of them were clinical trials: a double-blind, multicentre, phase III trials comparing clinical response, sustained clinical response, and safety of surotomycin and vancomycin (26, 27); a randomized, double-blind, dose-ranging, parallel group, phase II trial to evaluate the effects of surotomycin and vancomycin on CD and microbiota (28); a randomized, double-blind, placebo-controlled, phase I trial to characterize the safety, tolerability, and plasma pharmacokinetic profile of single and multiple ascending oral doses of surotomycin in healthy volunteers (29); a randomized, controlled, double-blind, non-inferiority, multicentre, phase II trial to evaluate the clinical response at end of treatment (30); and a double-blind, randomized, placebo controlled, multiple-dose phase I trial to evaluate the impacts of ascending doses of surotomycin on major organism groups in the gut microbiota of healthy volunteers (31).

Regarding ridinilazole, our literature search identified 10 studies, 2 of them were clinical trials: a randomized, double-blind, active-controlled, non-inferiority, phase II trial comparing the sustained clinical response (defined as clinical cure at the end of treatment and no recurrence within 30 days) of ridinilazole and vancomycin (32) and a double-blind, randomized, placebo-controlled, phase I trial to assess safety and tolerability of single and multiple oral doses of ridinilazole in healthy volunteers (33).

For LFF571, our literature search identified 16 studies, 3 of them were clinical trials: 2 multicenter, randomized, evaluator-blind, active-controlled, parallel-group, phase II trials to compare safety, efficacy and pharmacokinetics of LFF571 to those of vancomycin CDI (34, 35) and a randomized, double-blind, placebo-controlled, single- and multiple-ascending oral dose, phase I trial to determine the safety, tolerability, and pharmacokinetics of LFF571 in healthy subjects (36).

The literature search on CRS3123 and ramoplanin identified 10 and 28 studies, respectively.

For CRS3123, one clinical trial was identified, a double-blind, placebo-controlled, dose escalation, study to evaluate the safety and systemic exposure of CRS3123 after a single oral dose (37).

No clinical trials were identified for ramoplanin.

For fusidic acid, our literature search identified 40 studies, 2 of them were clinical trials: a randomized controlled, double-blind, phase III trial to compare the efficacy of fusidic acid and metronidazole for the treatment of patients with a first CDI episode (38) and a randomized controlled, double-blind, phase III trial to evaluate culture positivity for CD, development of resistance and association with treatment failure or recurrence of CDI after fusidic acid or metronidazole treatment (39).

For nitazoxanide and rifampin, 54 and 44 studies were identified, respectively.

For nitazoxanide, three trials were identified: a double-blind, randomized, controlled phase III trial to compare nitazoxanide with vancomycin for treatment of CDI (40); a preliminary, uncontrolled, open-label phase II study to evaluate response to nitazoxanide treatment in patients with CDI who failed a previous metronidazole treatment (41); and a randomized, double-blind, phase III trial to compare nitazoxanide to metronidazole for the treatment of CDI (42).

For rifampin, a clinical trial was identified; it was a prospective, randomized, single-blinded trial to compare therapy with metronidazole alone versus therapy with metronidazole and rifampin for 10 days to treat primary CDI (43).

The literature search on rifaximin identified 110 studies, 1 of them was a clinical trial. This trial was a randomized, double-blind, placebo-controlled, single-center pilot study to assess rates of recurrent diarrhea in patients with CDI who received rifaximin versus placebo immediately after the standard therapy (44).

The literature search on tigecycline identified 78 studies, none of them was a clinical trial.

The literature search on NVB302, thuricin CD, and lacticin 3147 identified 3, 8, and 3 studies, respectively.

The literature search on auranofin and acyldepsipeptide antimicrobials identified two and one study, respectively. No clinical trials were identified by the literature search on NVB302, thuricin CD, lacticin 3147, auranofin, and acyldepsipeptide antimicrobials.

Table 1 shows the main characteristics of the novel antimicrobials in development for CD, included their inhibition of CD sporulation and toxins production.

**Table 1:** Main characteristics and activity on CD spore and toxins production of the novel antimicrobials in development for CD.

Antimicrobials in development	Chemical structure description	Mode of action	Gut availability and effect on gut microbiota	Activity on CD sporulation and CD toxin inhibition	Selectivity against CD or narrow spectrum activity	MIC ranges against CD	Reference
Cadazolid	Oxazolidinone antimicrobial, containing a quinonolone pharmacophore incorporated in an oxazolidinone ring	Bacterial DNA and protein synthesis inhibition	Minimum observed fecal concentration following a single 3,000 mg oral dose from 24 h up to day 7 was 311 µg/g. Maximum daily individual fecal concentration after up to 7 days was 1,419 µg/g	Inhibited CD sporulation even at sub-growth-inhibitory concentrations	Narrow spectrum	Baseline MIC_50_, MIC_90_ and MIC ranges were 0.125 mg/L, 0.25 mg/L, and 0.06–0.25 mg/L, respectively	(24, 25, 47, 48, 51)

Surotomycin	13-Amino acid semisynthetic lipopeptide	Calcium-dependent cell membrane depolarizing agent	High excretion in feces, achieving high colonic concentrations	No	Bactericidal activity against Gram-positive bacteria. Not negligible activity on gut microbiota, including *Bifidobacterium* and *Lactobacillus* spp.	MIC_90_ 0.125–0.25 µg/ml in TY medium. The initial bacterial titer was 5 × 10^5^ CFU per ml	(28, 52–58)

Ridinilazole	Heterocyclic antibacterial	Bacterial DNA synthesis inhibition	>97% passes unchanged in the colon, achieving high concentrations at this site	Inhibits sporulation, as well as toxins A and B	Narrow-spectrum activity against Gram-positive pathogens including CD, minimally affecting the host gut microbiota	*In vitro* studies have reported MIC_90_ values of 0.125–0.25 µg/ml	(33, 65–69)

LFF571	Thiopeptide antibiotic	Bacterial protein synthesis disruption by inhibition of the elongation factor Tu	Low oral bioavailability, high colonic concentrations after oral administration	Reduce CD toxin production	activity against other Gram-positive anaerobes and Gram-positive aerobes, including *lactobacilli* and *enterococci*	MIC range of 0.06–0.5 mg/L	(34–36, 75–82)

Ramoplanin	Glycolipodepsipeptide antibiotic	Indirect inhibition of peptidoglycan biosynthesis	High colonic concentrations after oral administration	Inhibited *in vitro* spore counts at 300 µg/ml ramoplanin concentrations in feces	Activity against aerobic and anaerobic Gram-positive bacteria	MIC range of 0.25–0.50 µg/ml	(83, 84, 86–92)

CRS3123	Diaryldiamine	Inhibition of bacterial protein synthesis (bacterial methionyl-tRNA synthetase)	Not negligible systemic absorption after oral administration	At concentrations as low as 1 mg/L, CRS3123 decrease CD sporulation and inhibits *in vitro*toxin production	Activity against Gram positive bacteria including *Staphilococcus* and *Enterococcus*, sparing *Lactobacillus* and *Bifidobacterium*	MIC range of 0.5–1 mg/L and MIC_90_:1 mg/L	(94–96)

Fusidic acid	Polysaccharide	Inhibition of bacterial protein synthesis; it also acts as a blocker of the adhesion molecule L-selectin, involved in the inflammatory response to CD	Levels in feces correspond to 2% of the oral dose, around 0.3 mg/L after an oral dose of 250 mg. A significant intraluminal secretion due to inflammation may result in higher local therapeutic concentrations	n/a	Activity against S*taphylococci*, S*treptococcus*, and *Enterococcus* spp., anaerobic Gram-positive bacteria and Gram-negative anaerobic bacteria	MIC_50_: 0.75 mg/L and MIC_90_: 2 mg/L. MIC range of 0.125–4 mg/L	(38, 39)

Nitazoxanide	Nitrothiazole benzamide	Anaerobic metabolism inhibition	Two-thirds of the drug is excreted in the feces after oral administration	n/a	Activity against anaerobic bacteria, including *B. fragilis, Bifidobacterium* spp. and *Propionibacterium* spp.	Median MIC_50_: 0.5 µg/ml; MIC_90_: 1 µg/mL; MIC range of 0.25–2 µg/ml	(40, 42, 134, 165)

Rifampin	Rifamycin antimicrobial class	Inhibition of DNA-dependent RNA polymerase after binding to the beta subunit of the enzyme	Mostly systemically absorbed when given orally, peak serum concentrations of 7–10 µg/mL following a dose of 600 mg	n/a	Broad spectrum activity against gram-positive *Staphylococci, Enterococci*, gram-negative organisms	MIC_50_: 0.002 µg/ml and MIC_90_: 0.19 µg/ml. Potential risk of resistance development	(101)

NVB302	Type B lantibiotic	Inhibition of cell wall biosynthesis by binding lipid II	n/a	n/a	Wide range of Gram-positive bacteria. Not negligible impact on gut microbiota including Clostridia spp., *Bifidobacterium* spp., *B*. *fragilis, Enterococcus* and *Lactobacillus* spp.	n/a	(107)

Thuricin CD	Modified bacteriocin antimicrobial	Acts on bacterial membrane, causing the collapse of the membrane potential, membrane depolarization and cell death	n/a	n/a	Narrow spectrum activity against CD, minimal impact on gut microbiota	MIC_90_: 1,17 µg/ml	(90, 108, 109)

Lacticin 3147	Two-peptide molecule possessing intramolecular rings formed by the thioether aminoacids lanthionine and beta-methyllanthionine	Binding of the membrane-bound cell wall precursor lipid II and subsequent formation of a membrane pore and cell lysis	Oral administration is not feasible due to bacteriocins sensitivity to gastric proteolysis. the compound could be administered *via* the anal route	n/a	Broad-spectrum activity against Gram-positive bacteria, high impact on several phila of the gut microbiota	MIC range of 0.95–15 mg/ml	(106, 108, 110)

Auranofin	Gold complex containing a Au-S bond stabilized by a triethyl phosphine group [2,3,4,6-tetra-o-acetyl-1-thio-β-d-glucopyranosato-*S*-(triethyl-phosphine) gold]	Sequesters inorganic selenium, thus impairing CD selenium metabolism and seleno-proteins synthesis	High excretion in feces after oral administration	n/a	Specificity against CD	estimated IC_50_ values of 775–1,000 nM.	(111, 112)

Acyldepsipeptide-1		Acts inducing over-activation of intracellular caseinolytic ATP-dependent proteases, therefore disrupting protein metabolism in bacterial cell	n/a	Acyldepsipeptide-1 targets are related to the intracellular systems sigma factors σ (E) and MazEF, which play a key role in CD sporulation	Broad-spectrum activity against Gram-positive bacteria	n/a	(113–115)

Rifaximin	Rifamycin antimicrobial class	Inhibition of bacterial RNA synthesis	High colonic concentrations after oral administration	n/a	Broad-spectrum activity against gram-positive and enteric gram-negative pathogens, including *E. coli, Lactobacillus* spp.; despite its broad antibiotic spectrum, rifaximin appears to have little impact on normal gastrointestinal flora	MIC range of 0.004–128 µg/ml. Risk of emergence of resistance	(102, 148–150, 166)

Tigecycline	Glycylglycine class, structural analog of minocycline	Bacterial protein synthesis inhibitor	Tigecycline achieves high concentrations in the bile and gastrointestinal tract (median fecal concentration in human volunteers, 5.6 mg/kg on day 8 of administration)	Bacteriostatic activity against CD, prevents CD toxin production	Broad-spectrum activity against gram-positive and gram-negative bacteria, including *Fusobacterium* spp., *Prevotella* spp. and *Bacteroides fragilis*. Tigecycline causes less alteration on gastrointestinal flora than other broad-spectrum antibiotics	MIC range of 0.016–0.25 mg/L	(118–134, 138, 139, 142)

Table 2 shows the phase of the latest clinical trials for the novel antimicrobial in development for CD.

**Table 2:** Phase of the latest clinical trials for the novel antimicrobial in development for CD.

Antimicrobial in development	Phase of the latest clinical trials	References
Cadazolid	II	(24)
Surotomycin	III	(26, 27)
Ridinilazole	II	(32)
LFF571	II	(34, 35)
Ramoplanin	II	(92)
CRS3123	I	(37)
Fusidic acid	III	(38, 39)
Nitazoxanide	III	(40, 42)
Rifampin	II	(43)
NVB302	I	Unpublished
Thuricin CD	None	None
Lacticin 3147	None	None
Auranofin	None	None
Acyldepsipeptide-1	None	None
Tigecycline	None	None
Rifaximin	II	(44)

The main features of the novel anti-CDI molecules are described below.

### Cadazolid

Cadazolid, formerly known as ACT-179811, is a novel oxazolidinone antimicrobial characterized by a chemical structure containing a quinonolone pharmacophore incorporated in an oxazolidinone ring (45). The systemic bioavailability of cadazolid is negligible, as well as its absorption from the intestine following oral administration (46). The exact mechanism of action of cadazolid is multifaceted and not still fully elucidated; it has been shown that the quinolone pharmacophore of the cadazolid molecule inhibits both DNA and protein synthesis in the bacterial cell (45, 47). Upon administration, this agent leads to impaired bacterial protein synthesis and consequently to bacterial cell death.

Recently, Locher et al. adopted a macromolecular labeling assay to investigate the site of action of cadazolid in the bacterial cell (48). By monitoring cadazolid incorporation of labeled macromolecules, authors elucidated the cadazolid inhibitory action on the bacterial cell wall synthesis (48). Authors also demonstrated cadazolid influence on bacterial transcription and translation, by means of cell-free coupled transcription/translation assays (48).

Since cadazolid demonstrated to be highly active against CD *in vitro* as well as in gut and animal models, it has been proposed for the treatment of CDI (24, 49).

Interestingly, in contrast with its wide range of inhibitory effects on bacterial synthesis processes, in a human gut model, cadazolid demonstrated a narrow spectrum antimicrobial activity (50). In this model, cadazolid eliminated CD cells while having a very limited impact on the normal gut microbiota (50).

Cadazolid also showed potent biological effects on CD toxin and spore formation (45, 47, 48).

In an *in vitro* study comparing vancomycin and cadazolid effect on CD, whereas vancomycin failed to inhibit spore formation, cadazolid markedly inhibited CD sporulation even at sub-growth inhibitory concentrations (25, 49).

The narrow spectrum activity against CD, together with the ability to prevent sporulation, suggests that cadazolid has the potential to reduce CDI recurrence (25).

A single-center, open-label, single oral dose phase I study showed that cadazolid was well tolerated in patients with severe CDI following a single dose of 3,000 mg. Systemic exposure was very low and concentrations in feces were not only very high at peak but remained elevated for several days after single-dose administration (25).

The expectations on this compound have been also supported by a phase II trial reporting lower recurrence rates and higher sustained clinical response rates in patients with CDI treated with cadazolid as compared to those treated with vancomycin (24).

Preclinical and early clinical studies are therefore promising and demonstrate that cadazolid may be an effective option for the treatment of CDI. The results from the ongoing phase III trial will better define the role of cadazolid for the future CDI treatment.

### Surotomycin

Surotomycin, previously known as CB-183315, is an orally administered, water-soluble, 13-amino acid semisynthetic lipopeptide (51–56). It was originally obtained from daptomycin after a two-step process of enzymatic cleavage of the decanoyl side chain and its substitution with the (E)-3-(4-pentylphenyl)-but-2-enoyl residue in its molecular structure (56).

Not surprisingly, surotomycin mechanism of action is similar to daptomycin, it acts as a calcium-dependent cell membrane depolarizing agent (31).

Similarly to daptomycin, surotomycin possesses bactericidal activity against Gram-positive bacteria (57). The impact of surotomycin on gut microbiota is not negligible, with bactericidal activity against Gram-positive microbiota components including *Bifidobacterium* and *Lactobacillus* spp. but limited effect on Gram-negative species, including *Bacteroides* (57). An *in vitro* gut model confirmed limited effects on *Bacteroides fragilis* after surotomycin administration in comparison with vancomycin (58).

Surotomycin has shown to be effective in CDI animal models (59). A study reported similar survival rate of CD infected hamsters treated with surotomycin or with orally administered vancomycin (59).

Surotomycin proved its efficacy also in human gut models (57, 58, 60, 61).

In summary, evidences from *in vitro* and *animal* studies on surotomycin showed a potent *in vitro* effect on CD and an efficacy to treat CDI similar to that of vancomycin; however, a more sparing effect on gut microbiota has been reported for surotomycin (58, 62).

Preliminary studies on orally administered surotomycinin in humans reported a minimal systemic bioavailability (<1%) and a high excretion in feces, achieving high colonic concentrations (29). Minimal systemic effects have been observed during phase I trials after oral administration (29, 57).

A randomized, double-blind, multicenter phase II trial including 209 CDI patients who received either surotomycin 125 or 250 twice a day for 10 days or vancomycin 125 mg four times daily for 10 days was performed (30). Cure rates at the end of treatment were similar for surotomycin and vancomycin, while recurrence rates and sustained cure rates were higher for surotomycin than for vancomycin (30).

Therefore, two parallel phase III clinical trials were started to demonstrate surotomycin non-inferiority to vancomycin for resolving CDI and surotomycin superiority in preventing recurrence (26, 27).

Unfortunately, the first phase III trial evaluating surotomycin 250 mg twice a day efficacy against CDI in comparison with vancomycin 125 mg four times a day did not meet the study endpoints (27).

Furthermore, a parallel phase III trial was conducted with the primary objective to demonstrate the non-inferiority of surotomycin versus vancomycin in response rates at the end of treatment in adults with CDI. As secondary objectives, this trial aimed to assess clinical response over time and sustained clinical response superiority of surotomycin compared with vancomycin (26). This trial randomized 608 patients to receive twice daily surotomycin 250 mg for 10 days or vancomycin 125 mg four times daily for 10 days (26).

The primary endpoint of the trial was met, and surotomycin demonstrated non-inferiority to vancomycin for the treatment of adults with CDI (clinical response rate at the end of treatment: 83.4% *vs*. 82.1%) (26). However, surotomycin failed to demonstrate a significant benefit over the existing vancomycin therapy (26).

In conclusion, although surotomycin was generally well tolerated during the conduction of the phase III trials, the published results make doubtful that surotomycin will be introduced for the treatment of CDI (63).

### Ridinilazole

Ridinilazole, formerly known as SMT19969, is a new narrow-spectrum synthetic antibiotic (64, 65). The mechanism of action of this novel class of heterocyclic antibacterials has not been fully elucidated, but we know that ridinilazole inhibits DNA bacterial synthesis (64). Moreover, Bassères et al. demonstrated that ridinilazole induces CD cell elongation while inhibiting sporulation, in contrast to other traditional anti-CD antimicrobials (66).

Pharmacokinetics of ridinilazole appears ideal for the treatment of CDI, as it is non-systemically absorbable after oral administration and more than 97% of the antimicrobial passes unchanged in the colon, achieving high concentrations at this site (33).

Ridinilazole showed a narrow-spectrum activity against Gram-positive pathogens, including CD and it minimally affects the host gut microbiota (67–69).

Interestingly, ridinilazole specifically inhibits the growth of CD and has no effect on other *Clostridia* species; this could have a beneficial effect on the imbalance between CD and *Clostridia scindens* that has been demonstrated to have an important role in CDI development (67).

*Clostridia scindens* is one of the few bacterial species able to convert primary bile salts into secondary bile salts in the human gut. A bile acid-dependent, *C. scindens*-mediated CDI inhibition model has been recently hypothesized (70–72). According to this model, microbiota-mediated modification of bile acids contributes to host resistance to intestinal pathogens such as CD (70–72). In health persons, a microbial network in the gut provides resistance against CDI; exposure to broad-spectrum antibiotics leads to intestinal microflora disruption, including a reduction in *C. scindens* population. This imbalance between CD and *C. scindens* gut colonization prevents the metabolism of bile acids increasing the ratio between primary and secondary bile acids, this in turn facilitates CD germination and overgrowth (70–73). Among commensal microbiota components, C. *scindens* is one of the bacterial species having the ability to convert primary bile salts into secondary bile salts, promoting inhibition of CD vegetative growth. The specific activity of ridinilazole against CD, sparing other clostridia species, may be therefore a promising feature.

In preclinical studies adopting the CD-infected hamster model, ridinilazole has shown to be effective, with an observed survival rate similar to that of vancomycin and fidaxomicin (68, 69).

After the completion of a phase I safety study demonstrating that the compound is well tolerated when assumed orally at a dosage up to 2 g a day over 10 days (33), a phase II double-blind trial (CoDIFy) randomized 100 CDI patients to receive either ridinilazole 200 mg bid or vancomycin 125 mg qid (32).

The trial results demonstrated ridinilazole superiority in achieving response rates at the end of treatment (77.8% and 69.7% for ridinilazole and vancomycin, respectively), in reducing rates of recurrent CDI (14.3% and 34.8% for ridinilazole and vancomycin, respectively) and in obtaining sustained clinical responses (66.7% and 42.4% for ridinilazole and vancomycin, respectively) (32). These results sound promising and support larger phase III clinical trials.

The results from a recently completed randomized trial (NCT02784002) comparing ridinilazole to fidaxomicin for the treatment of CDI have not yet been published (74).

### LFF571

LFF571 is a novel semi-synthetic cyclic lipopeptide antibiotic derived from a natural metabolite produced by the actinomycete *Planobisporarosea* (75, 76). It belongs to a new class of thiopeptide antibiotics and acts disrupting bacterial protein synthesis by inhibiting the elongation factor Tu (EF-Tu), a bacterial factor involved in peptide synthesis (75–78).

LFF571 showed interesting pharmacokinetics features for the treatment of gastrointestinal infections, as it possesses low oral bioavailability and reaches high colonic concentrations (35, 36).

*In vitro* studies demonstrated LFF571 activity against CD, with a minimal inhibitory concentration (MIC) (79–82). LFF571 also possesses activity against other Gram-positive anaerobes and some Gram-positive aerobes, including *lactobacilli* and *enterococci* (79–81).

A study performed in the CDI hamster models showed that LFF571 administration prevented CDI associated mortality (82).

Subsequently, a placebo-controlled phase I trial assessing LFF571 safety and tolerability demonstrated that a single LFF571 dose up to 1,000 mg and repeated doses up to 200 mg four times a day for 10 days were safe and well tolerated in healthy volunteers (36).

Finally, in 2015, a phase II trial has been carried out to compare LFF571 and vancomycin safety and efficacy (34). This evaluator-blind trial randomized 72 patients with moderate severity CDI to receive a 10 days course of either LFF571 200 mg four times daily or vancomycin 125 mg four times daily (34). The trial results showed higher clinical response rates at the end of treatment with LFF571 (90.6% *vs*. 78.3%), unfortunately also higher recurrence rates were reported with LFF571 (37% vs 31%) (34).

No further phase II or phase III trials for LFF571 have been reported so far.

### Ramoplanin

Ramoplanin is a glycolipodepsipeptide antimicrobial that exerts its mechanism of action preventing cell wall peptidoglycan biosynthesis (83). More precisely, ramoplanin indirectly inhibits the transglycosylases responsible for peptidoglycan biosynthesis by sequestering their intermediate substrate Lipid II at the interface between the extracellular environment and the bacterial membrane (83). Binding to the key intermediate moiety lipid II, ramoplanin leads to the disruption of bacterial wall and therefore bacterial death (83, 84).

This compound is non-absorbable orally and achieves high colonic concentrations (85).

Ramoplanin has activity against both aerobic and anaerobic Gram-positive bacteria, including vancomycin-resistant *Enterococcus* (86, 87).

Regarding its activity against CD, an *in vitro* model clearly showed that ramoplanin molecule can bind CD spores and also kills vegetative CD cell with high efficacy (87–91).

Ramoplanin activity against CDI has been demonstrated also in animal models (86).

Interestingly, ramoplanin superiority over vancomycin in reducing CD sporulation and spore release has been demonstrated in animal model showing that CD spores were less often recovered from the ramoplanin-treated hamsters as compared to those treated with vancomycin (86).

Subsequently, in a phase II trial, 86 CDI patients were randomized to receive either ramoplanin, 200 mg twice daily or 400 mg twice daily, or vancomycin, 125 mg four times daily for 10 days. The two arms receiving ramoplanin showed similar clinical cure rates at the end of treatment (83% and 85%, respectively, in comparison to 86% of vancomycin), but also higher rate of adverse events, with nausea, vomiting, and diarrhea as the most frequently reported adverse events in the ramoplanin arms (85, 92).

Nonetheless, in the light of the promising features of this drug, a phase III trial on ramoplanin against CDI has been planned and has been recently approved by the FDA (85, 92, 93).

### CRS3123

CRS3123, formerly known as REP3123, is a recently developed fully synthetic diaryldiamine antimicrobial (93).

CRS3123 prevents both CD growth and CD spore production by inhibiting bacterial protein synthesis (85, 94, 95). Interestingly, this antimicrobial acts on the bacterial methionyl-tRNA synthetase, has limited effect on the structurally distinct methionyl-tRNA synthetases of Gram-negative bacteria and humans (85, 94, 95), and possesses activity against CD and other Gram positive bacteria including *Staphylococcus* and *Enterococcus*. However, CRS3123 is inactive against major intestinal Gram-positive colonizers, including *Lactobacillus* and *Bifidobacterium* (95, 96).

Following the evidences of CRS3123 efficacy for CDI treatment obtained from the hamster model (94), the results from a phase I study to assess the safety, tolerability, and systemic exposure of escalating doses of CRS3123 in humans have been recently published (37).

In this single-center, double-blind, placebo-controlled phase I trial, escalating doses of CRS3123 were administered orally to the study participants. The study enrolled 40 participants randomized to receive study product or placebo, with a CRS3123 dose range of 100, 200, 400, 800, and 1,200 mg (37).

Reported adverse events were similar in severity and frequency for participants who received active drug and for those who received placebo, and all the adverse events in the study drug group were mild or moderate (37).

The bioavailability of CRS3123 following oral administration could not be accurately assessed during this trial, because of the absence of standards for the metabolites. Nonetheless, a not negligible fraction of the administered oral dose of CRS3123 was detected systemically (37).

Phase II trials are urgently needed in order to assess CRS3123 efficacy for CDI in humans.

### Fusidic Acid

Fusidic acid is a relatively well-known antimicrobial belonging to the class of polysaccharides and originally developed from the fungus *Fusidium coccineum* (93).

Fusidic acid works by interfering with bacterial protein synthesis, by preventing the translocation of the elongation factor G from the ribosome (93). Of interest for its implication in CDI treatment, fusidic acid also acts as a blocker of L-selectin, an adhesion molecule involved in the inflammatory response to CD (93).

Concerning the economic cost for a course of CDI treatment, fusidic acid may represent one of the cheapest options in US, with a cost similar to metronidazole (38).

So far, a phase III trial by Wullt et al. has been published on fusidic acid for CDI treatment (38, 39). The authors performed a prospective, randomized-controlled, double-blind trial on 131 patients to compare the efficacy of fusidic acid 250 mg orally three times to that of metronidazole 400 mg orally three times daily for 7 days for initial CDI episodes (38). In the fusidic acid group, clinical cure at the end of treatment was achieved in 83% of patients in comparison to 93% in the metronidazole group (*P* = 0.116) (38). Clinical CDI recurrence was described in 27% and 29% of patients receiving fusidic acid and metronidazole, respectively (39).

Of note, in the group of patients treated with fusidic, the emergence of fusidic acid resistance was reported in the 55% of the CD infecting strain (39). The mechanisms of fusidic acid resistance in CD are still unknown but the emerging fusidic acid resistant CD strains may easily be transmitted between patients, hampering any future wide-spread use of fucidic acid for CDI (39). Nevertheless, fusidic acid monotherapy may still represent a possible future option for selected settings, i.e., units with a high rate of vancomycin-resistant enterococci intestinal colonization or with CDI patients who can not tolerate the standard CDI treatment (39).

At present, there are no further phase III trials on the development of fusidic acid as anti-CDI treatment (93).

### Nitazoxanide

Nitazoxanide is a nitrothiazole benzamide that was originally approved as an anti-parasitic drug, but over time, it also showed activity against bacterial enteric pathogens, including CD (97).

After oral administration, nitazoxanide is mostly excreted in feces, and it has been well tolerated in the studies performed in humans, with no reports of serious adverse events, although rare occurrence of elevated creatinine levels and alanine aminotransferase in serum has been reported (98, 99).

So far, clinical trials have compared nitazoxanide use for CDI with both vancomycin and metronidazole.

A preliminary, uncontrolled, open-label phase II study on 35 CDI patients who failed metronidazole treatment for a first CDI episode or who had recurrent CDI assessed a 74% response rates after a 10-day nitazoxanide treatment, but also a 33% recurrence rate (41).

Subsequently, in a phase III, double-blind trial, 110 primary CDI patients were randomized to receive a 10-day treatment course with nitazoxanide or metronidazole, reporting similar clinical response rate at the end of treatment (89.5% and 82.4% for nitazoxanide and metronidazole, respectively) and similar CDI recurrence rates (13.9% and 24% for nitazoxanide and metronidazole, respectively) (42).

Regarding nitazoxanide comparison with vancomycin, a prospective, double-blind randomized trial was conducted in CDI patients (40). The trial compared the efficacy of 10 days of oral nitazoxanide therapy versus 10 days of oral vancomycin in CDI patients (40). Unfortunately, this trial was prematurely stopped for unclear reasons, with a total number of 49 enrolled patients. Even if similar clinical cure rates at the end of treatment (77% for nitazoxanide and 74% for vancomycin) and recurrence rates (5% for nitazoxanide and 7% for vancomycin) were observed, these results did not reach statistical significance (40).

However, nitazoxanide still shows promising features as a future treatment of CDI, and recently its use for severe recurrent cases of CDI has been reported (100).

### Rifampin

First synthesized in 1965, rifampin is a well-tolerated antimicrobial compound which exerts its activity by inhibiting bacterial RNA synthesis (101). Although rifampicin cannot be considered a novel antimicrobial, over time its activity against CD leaded to consider its use for CDI treatment (102, 103).

However, in a phase II trial testing rifampin in association with metronidazole, similar initial cure rates were found between rifampin plus metronidazole versus metronidazole alone (63% and 65%, respectively), and the study was ended prematurely because of the non superiority of the combination therapy (43).

A different phase III trial was begun in 2008 and was recently completed (NCT00182429), but no results have been reported so far (93).

Moreover, beside all the uncertainties on its efficacy as a single agent against CDI, the emergence of resistance to rifampin represents a further matter of concern (104, 105).

### NVB302

The antimicrobial NVB302 was first isolated from *Actinoplanes liguriae* (93, 106).

After promising pre-clinical studies in human gut models (107), no results have been published from phase I trials, and no development of phase II or III trials has been reported (106, 107).

### Thuricin CD

Thuricin CD is a recently developed, modified bacteriocin antimicrobial, which exhibited excellent narrow spectrum activity against CD (108, 109).

This compound is showing promising features; interestingly, in a human gut research model, it displayed a minimal impact on gut microbiota, sparing *Firmicutes, Bacteroides*, and *Proteobacteria* in comparison with traditional anti-CD antibiotics (109).

At present no trials have been conducted on Thuricin CD, and its safety and efficacy for CDI are still to be demonstrated (106).

### Lacticin 3147

Lacticin 3147 is a small 2-peptide molecule “lantibiotic” synthesized by *Lactococcus lactis* (110). Even if it has been demonstrated a high activity of this molecule against CD, its broad-spectrum antimicrobial activity against Gram-positive bacteria and its high impact on several phila of the gut microbiota make unlikely lacticin 3147 future use for CDI treatment (106, 108, 110).

### Auranofin

Auranofin is a molecule originally approved for the treatment of rheumatoid arthritis (111). This compound gained attention for a possible treatment in CDI because of its potent *in vitro* inhibitory activity of CD and its considerable excretion in feces after oral administration (111).

Auranofin acts on CD by sequestering inorganic selenium, thus impairing CD selenium metabolism and seleno-proteins synthesis, essential for the bacterium survival (112).

Interestingly, this mechanism of action should confer to auranofin specificity or quasi-specificity against CD.

At present, despite these promising features, no clinical trials have been published on auranofin use for CDI.

### ADEP-1

ADEP-1 (acyldepsipeptide-1) is a novel bactericidal antimicrobial molecule belonging to the acyldepsipeptide antibiotic class (113). These natural antibiotics are originated from the soil bacteria *Streptomycetes* (113). More precisely, ADEP-1 originates from the bacteria *Streptomyces hawaiiensis* and showed a potent activity against Gram-positive bacteria (114).

As other acyldepsipeptide antibiotics, ADEP-1 acts disrupting the protein metabolism in bacterial cell, by inducing over-activation of intracellular caseinolytic ATP-dependent proteases (114). Subsequently, the intracellular protein metabolism derangement causes cell division, differentiation, and sporulation impairment, finally leading to bacterial cell death (114, 115).

Interestingly, it has been shown that the CD proteases targeted by acyldepsipeptide antimicrobials are also related to the intracellular systems sigma factors σ (E) and MazEF, which play a key role in CD sporulation (114, 116).

Recently, starting from the consideration of ADEP-1 Gram-positive bactericidal activity and the presence in CD of caseinolytic proteases, potential target of this compound, some authors proposed the use of ADEP-1 and other acyldepsipeptide antimicrobials for the treatment of CDI, alone or in combination with other antimicrobial classes (114).

Even if these authors highlighted the lack of specificity against CD of this compound, in our opinion, the promising feature of ADEP-1, including its bactericidal activity and its potential effect on CD sporulation, urges the design of pre-clinical studies on this molecule.

At present, there are no ongoing clinical trials on acyldepsipeptide antibiotics for CDI treatment.

### Tigecycline

Tigecycline is a broad-spectrum antimicrobial of the glycylglycine class (117). It acts as a protein synthesis inhibitor, with activity against Gram-positive, Gram-negative and anaerobe bacteria, including *Fusobacterium* spp., *Prevotella* spp., *Porphyromonas* spp., and *Bacteroides fragilis* (117, 118).

Tigecycline has been approved for treatment of complicated skin infections and complicated intra-abdominal infections (119).

Tigecycline is not registered for use in CDI; however, it exerts a bacteriostatic activity against CD (119–121).

Importantly, tigecycline achieves high concentrations in the bile and gastrointestinal tract after intravenous administration, with a median fecal concentration in human volunteers of 5.6 mg/kg after 8 days, and has been proposed as an alternative agent for the treatment of CDI in humans (122).

A large pan-European study, conducted between 2011 and 2014 across 22 European countries, reported that tigecycline had *in vitro* activity against all the CD isolates (2,830) tested for tigecycline susceptibility, with a mean MIC of 0.04 mg/L (123).

Moreover, multi-drug resistant CD strains have been found susceptible to tigecycline, showing a MIC range from 0.016 to 0.25 mg/L (118, 123–135).

Therefore, tigecycline represents a potential antibiotic treatment for CDI (118, 125, 135–137).

Interestingly, the effects of tigecycline on sporulation and toxin production have been evaluated by means of *in vitro* experiments and animal models, demonstrating that tigecycline is effective in preventing CD overgrowth and CD toxin production (118, 128, 129, 138).

Regarding the impact on gut microbiota, there is evidence that tigecycline causes a significant alteration of the microbiota, including a reduction of *Bacteroides spp* (118, 132, 139–141). However, Jump et al. demonstrated that, in comparison to other broad-spectrum antibiotics, the use of tigecycline is associated with a lower risk of alteration of colonization resistance to CD (142). In the study, levels of bacterial metabolites in fecal specimens from a mouse model were measured, showing that tigecycline treatment caused a less profound alteration of fecal metabolites in comparison to linezolid, piperacillin/tazobactam, and ceftriaxone (142).

A retrospective cohort study comparing 45 patients receiving tigecycline monotherapy to 45 patients receiving standard treatment for CDI, reported higher clinical cure rates with tigecycline than with vancomycin and metronidazole (143).

However, retrospective studies evaluating the efficacy of tigecycline adjunctive therapy for CDI reported conflicting results (143–147).

Moreover, three retrospective cohort studies failed to demonstrate a beneficial effect of adjunctive tigecycline CDI treatment on patients outcome in comparison to standard treatment alone (145–147).

A phase II trial started in 2011 could have been able to elucidate the role of tigecycline for CDI treatment but unfortunately was discontinued because of a too slow enrollment rate (NCT01401023) (119).

No further phase II or III trials have been started so far (93, 119).

Therefore, randomized controlled trials are needed to fully elucidate tigecycline efficacy and safety for the management of CDI.

### A Room for Preventing CDI Recurrence

#### Rifaximin

Rifaximin is a well-tolerated antimicrobial compound which belongs to the rifamycin class. Similarly to rifampin, rifaximin acts inhibiting bacterial RNA synthesis (117, 118, 148, 149).

Interestingly, rifaximin is minimally absorbed after oral administration, achieving high colonic concentrations (148–150).

Therefore, rifaximin shares with rifampin several interesting features, i.e., a potent activity against CD, but also several drawbacks, including the high risk of emergence of resistance (93, 151).

It was introduced for the treatment of traveler’s diarrhea, rifaximin has also been proposed for CDI treatment (148–150).

Initially, rifaximin has been tested as a “chaser” to augment vancomycin efficacy in the CDI treatment (148–150), however, rifaximin ability to spare enteric microbiota makes it a potentially useful agent for the prevention of CDI recurrence after a first CDI episode.

On this issue, three case series have investigated the use of rifaximin to prevent CDI recurrence so far (152–154).

The first case series reported the administration of a 2-week regimen of rifaximin immediately after the vancomycin standard treatment in eight CDI patients with multiple recurrences (152). During the follow-up after this protocol, only one out of the eight patients experienced CDI (152).

In a report by Garey et al, five patients with multiple CDI recurrences received a rifaximin tapering regimen for 4 weeks (154). None of these subjects experienced CDI recurrence after the rifaximin protocol during a follow-up of at least 54 days (154).

Afterward, a phase II, double-blind, randomized placebo-controlled study was carried out to assess rifaximin efficacy in preventing recurrences in 68 CDI patients (44). Immediately after the standard metronidazole or vancomycin based therapy, the treatment arm received rifaximin at the dose of 1.2 g daily for 20 days (44). The observed rate of recurrence resulted lower in the rifaximin arm in comparison with the placebo (15% *vs*. 31% of recurrence, respectively), but unfortunately the findings of the study did not reach a significant difference (44).

A new trial to evaluate a rifaximin tapering regimen in a larger cohort of CDI recurrences patients has been recently completed, and results have yet to be published (NCT01670149, available from http://clinicaltrials.gov).

## Discussion and Conclusion

In comparison with the traditional anti-CDI antimicrobial treatment, some novel antimicrobials reviewed in this study offer several advantages.

The favorable pharmacokinetic and pharmacodynamic profile, the narrow-spectrum activity and the specificity against CD that implicate a low impact on the gut microbiota composition, and the inhibitory activity on CD sporulation and toxins production are among the most promising features of these compounds.

First, the potential capacity of the new medication to reduce CDI recurrences preserving the human gut microbiota is of major importance.

In fact, beside well-known risk factors for the development of CDI, such as decreased stomach acidity, advanced age, renal disease, and other comorbidities, it has been recently demonstrated that the disruption of the intestinal microbiota is a key factor for CDI development (155–158). Microbiota disruption facilitates CD germination and overgrowth and therefore CDI development (155–158).

Previous studies have demonstrated that the microbiota of CDI patients is characterized by a decrease in species richness, with an association between loss of *Bacterioides, Lachnospiraceae*, and *Ruminococcaceae* and the development of CDI (156–158).

A broad spectrum antibiotic treatment given for concomitant infection, usually inactive against CD but very active against bacterial intestinal colonizers, represents one of the main causes of microbiota disruption.

Importantly, also the traditional anti-CD antimicrobial compounds may negatively impact on the indigenous gut flora, paradoxically facilitating the imbalance between “protective” enteric pathogens and CD (159).

In fact, metronidazole possesses bactericidal activity against the protective anaerobic philia of *Bifidobacterium* and *Bacteroides* (159); vancomycin has remarkable activity against *Enterococcus* spp. and *Bacteroides* (160, 161).

Also fidaxomicin, even if may be considered an advancement in terms of specificity against CD in comparison with metronidazole and vancomycin, has shown relevant activity against *Bacillus spp*., *Enterococcus* spp., *Lactobacilli*, and *Bifidobacterium* (162, 163).

The imbalance determined in the gut flora mediated by these traditional first-line anti-CD antimicrobials can subsequently facilitate CDI recurrence (159).

Interestingly, it has been observed that the period of major vulnerability for CDI recurrence starts from the end of the traditional anti-CDI treatment, when sub-inhibitory levels of CDI antibiotics are still present in the human gut and further contribute to a misbalance in the gut microflora (157, 163, 164). Therefore, despite the administration of these traditional antimicrobials, CD spores can survive in the gut and be facilitated to germinate by the microbiota disruption, leading to recurrent CDI and trapping the patient in a vicious “recurrent CDI cycle” (157, 163, 164).

In the light of these considerations, the potential narrow-spectrum activity and the specificity against CD showed by some novel antimicrobials in development for CDI appears of crucial importance, allowing to preserve microbiota integrity and thus reducing CDI recurrences.

The novel antimicrobials that better fit with this characteristic are cadazolid, surotomycin, ridinilazole, auranofin, and thuricin CD.

Second, the ecological impact of the new antimicrobials against CD has to be highlighted.

The activity against CD spores and the ability to prevent CD sporulation displayed by several novel antimicrobials suggest that these compounds may not only reduce CDI recurrence rate, but may also potentially reduce CD environment persistence, thus reducing CD spread in the hospital setting and CDI healthcare associated acquisition.

Cadazolid, ridinilazole, CRS3123, ramoplanin, and, potentially, the acyldepsipeptide antimicrobial are, among the antimicrobial reviewed in this paper, the most active compounds in reducing spore production (Table 1).

Along with the potential benefit of new anti-CDI antimicrobials, it is important to emphasize that some new molecules present limitations that could affect the probability of their approval for the CDI treatment.

Main drawbacks include the occurrence of CD resistant strains for rifampicin and fusidic acid; the failure to achieve a significant benefit over the existing traditional antimicrobial CDI treatment for surotomycin; the failure to reduce the CDI recurrence rate at the end of treatment for LFF571, and the relatively high impact on several phila of the gut microbiota for ramoplanin.

In conclusion, the novel antimicrobial molecules under development for CDI present promising key features and advancements in comparison to the traditional anti-CDI antimicrobials. Hopefully, in the near future, these new molecules will be effective alternatives to fight and prevent CDI, a condition which may actually represent a real “spiral of disease.”

## Author Contributions

All authors participated in writing the manuscript, revising it, and gave the final approval for publishing the manuscript.

## Conflict of Interest Statement

GG and MC declare no conflicts of interest. NP received consulting fees from MSD, Pfizer, Astellas, Zambon, Angelini, Gilead, Achaogen, The Medicine Company, 3 M, Beckton & Dickinson.
